# Influence of air pollutants on varicella among adults

**DOI:** 10.1038/s41598-021-00507-z

**Published:** 2021-10-25

**Authors:** Zixuan Wang, Xiaofan Li, Ping Hu, Shanpeng Li, Jing Guan, Bingling Wang, Feng Yang, Dongfeng Zhang

**Affiliations:** 1 Department of Epidemiology and Health Statistics, The School of Public Health, Qingdao University, No. 308 Ningxia Road, QingdaoShandong, 266021 China; 2 Qingdao Municipal Center for Disease Control and Prevention of Qingdao, Qingdao, Institute of Preventive Medicine, Qingdao, 266034 Shandong China

**Keywords:** Environmental impact, Viral infection

## Abstract

Little attention has been paid to the relationship between air pollutants and varicella among adults. We used data collected in Qingdao, China from 2014 to 2019. A combination of quasi-Poisson generalized linear model (GLM) and distributed lag non-linear model (DLNM) was applied to evaluate the association between exposure to air pollutants and varicella. And the effects of exposure to extremely high concentration (at 97.5th percentile) and low concentration (at 2.5th percentile) of air pollutants on varicella were also calculated. The level II of GB3095-2012 was used as the reference. A 10 μg/m^3^ increase of PM_2.5_ was significantly associated with an increased risk of varicella (lag day: 4, 5 and 6). The negative associations were found for NO_2_ per 10 μg/m^3^ increase from lag 15 to 19 day. The high PM_2.5_ concentration (135 μg/m^3^) was significantly associated with the increased risk of varicella (lag day: 6, 7). For NO_2_, the negative association was found at high concentration (75 μg/m^3^) on lag 15 to 20 day; and the positive relationship was shown at low concentration (10 μg/m^3^) on lag 15 to 20 day. Exposure to PM_2.5_ and NO_2_ were significantly associated with the risk of varicella among adults.

## Introduction

Varicella (also known as chickenpox) is an airborne disease caused by the varicella-zoster virus (VZV)^1,2^. In China, the average annual reported incidence rate of varicella in the past 2016–2019 was 55.05/100,000, which was much higher than that in 2005–2015 (23.04/100,000)^3^. The incidence rate of varicella increased year after year during 2016–2019 (from 35.50/100,000 to 70.14/100,000), and so is it in adults^4^.

Varicella is a common acute infectious disease in childhood, and is generally benign and self-limiting^5,6^. In adults, the incidence rate of varicella is considerably lower than that in children. Even though the incidence rate of varicella in adults is not high, severe complications including pneumonia, encephalitis, and even death can occur among adolescent and adult patients, especially in the first infection in adulthood^7,8^. In Qingdao, varicella vaccine for target age children is included in the expanded immunization program. Compared with children, varicella vaccine for adults is outside the scope of immunization program. Therefore, Varicella is still considered a critical public health issue worldwide, with about 4.2 million severe varicella complications and approximately 4200 related deaths per year estimated by the World Health Organization (WHO) in 2014^9^.

Ambient pollution has become a serious global concern for its harm to human health^10^. It have reported that exposing to air pollutants could adversely affect some chronic diseases^11–13^ and infectious diseases such as tuberculosis^14^, mumps^15^, hand-foot-mouth disease^16^, measles^17^, etc. Air pollution was proven to make humans more susceptible to the invasion of pathogens, then increasing the chance of the infection of respiratory virus^18^. For this reason, air pollution may also be related to the incidence of varicella.

To our best knowledge, there is no report about the association between other air pollutants expect PM_10_ and varicella. Only one study conducted by Yu et al. found that high PM_10_ was associated with the increase risk of varicella^19^. It is believed that other particulate matter such as PM_2.5_ and NO_2_ (nitrogen dioxide) are the important exposures to infectious diseases^20,21^. Besides, the study focusing on adult varicella was scarce. Due to adult varicella is often more serious, it is particularly important to study the varicella among adults. In the present study, we investigated the relationship between PM_10_, PM_2.5_, NO_2_, SO_2_ (sulfur dioxide), O_3_ (ozone) and the incidence of varicella among adults (≥ 20 years old) in Qingdao, eastern China during 2014–2019.

## Methods

### Study region

Qingdao (36°04′N 120°23′E) is located between longitude 119°30'E to 121°E and latitude 35°35'N to 37°09'N in Shandong Province, China (Supplementary Fig. 1). This coastal city has a total area of 11,293 square kilometers and the population is above 9.3 million. The climate of Qingdao is characterized as temperate monsoon and influenced by marine environment.

### Data collection

We extracted the daily varicella cases from 1 January 2014 to 31 December 2019 from China National Notifiable Disease Surveillance System (NDSS). Generally, varicella can be diagnosed by doctors according to fever and characteristic rash. In Qingdao, medical institutions must report varicella cases to local Center for Disease Control and Prevention (CDC) through NDSS within 24 h after diagnosis and complete case review.

The daily (24-h) pollutant data were collected from Qingdao ecological environment monitoring center from 1 January 2014 to 31 December 2019. There are nine state controlling air sampling sites to monitor the concentrations of air pollutants in Qingdao. The average daily concentrations of PM_10_, PM_2.5_, NO_2_, SO_2_ and maximum 8-h moving average ozone concentration (O_3_-8 h) of nine sites were used as the exposures in our analysis.

Meteorological information was downloaded from Meteorological Data Sharing Service System of China (http://data.cma.cn/), including daily cumulative precipitation (mm), average temperature (℃), average wind velocity (m/s), average atmospheric pressure (hPa), sunshine duration (h), and average relative humidity (%).

### Patient and public involvement

Our research was a type of ecological research, and only daily meteorological factors and the number of daily varicella cases were included in our study. The number of adult varicella cases was acquired from NDSS, without patients’ personal information. Patients did not participate in our study.

### Statistical analysis

The temporal distributions of all air pollutant variables and varicella cases were described by time series plots. Spearman’s correlation analyses were used to assess the correlations between meteorological factors and air pollutant variables. To address the potential multicollinearity, only variables whose r was < 0.7 can enter our model^11^.

In ecological research, the delayed effects were often shown in the effects of air pollutants on health outcomes, and the exposure–response curves are often nonlinear^14,22^. Thus, we adopted distributed lag nonlinear model to explore the relationship between air pollutants and varicella incidence^23^. Considering the varicella cases can be regarded as small probability events, we applied a combination of quasi-Poisson generalized linear model (GLM) and distributed lag non-linear model (DLNM) in our study^24^.

Firstly, a basis model without air pollutants was built. We chose natural cubic spline (ns) to restrict the influence of seasonality and long-term trend, and we used a degree of freedom (df) of 7/year^25,26^. To control the impacts of meteorological factors, ns functions with 3 df empirically were also adopted in our analysis^15,27^. Other covariates such as day of week (DOW) and public holiday in China were included in models.

Secondly, we established cross-basis functions to describe the bi-dimensional exposure-lag-response relationships. For exposure–response relationships, we adopted linear functions. And for lag space, the ns function with three internal knots placed on a logarithmic scale with equidistant values was used. The number of the internal knots was selected according to the minimum Generalized Cross Validation (GCV) score. Given the incubation period of varicella, we selected 20 days as maximum lag period in our research.

The single-pollutant model was:

$$\begin{gathered} {\text{Y}}_{{\text{t}}} \sim {\text{ quasi}} - {\text{Poisson}}\left( {\mu_{{\text{t}}} } \right) \hfill \\ {\text{Log }}\left( {\mu_{{\text{t}}} } \right) = {\text{ cb }}\left( {{\text{X}};{\text{ lag}},{ 3}} \right) \, + {\text{ ns }}\left( {{\text{time}},{\text{ df }} = { 7}/{\text{year}}} \right) \, + {\text{ ns }}({\text{winds}},{\text{ df }} = { 3}) \, + {\text{ ns }}\left( {{\text{humidity}},{\text{ df }} = { 3}} \right) \, + {\text{ ns }}({\text{precipitation}},{\text{ df }} = { 3}) \, + {\text{ ns }}\left( {{\text{temperature}},{\text{ df }} = { 3}} \right) \, + {\text{ ns }}\left( {{\text{sunshine}},{\text{ df }} = { 3}} \right) \, + {\text{ DOW }} + {\text{ holiday }} + {\text{ intercept}} \hfill \\ \end{gathered}$$

where t indicates the number of observation days; Y_t_ is the actual number of adult varicella cases on day t; μ_t_ represents the estimated daily adult varicella cases on day t; cb() represents the cross-basis function fitting the complex exposure-lag relationships for PM_10_, PM_2.5_, NO_2_, SO_2_, and O_3_-8 h, respectively; “lag,3” in cb() indicates 3 internal knots placed on a logarithmic scale with equidistant values for lag space; df means degree of freedom; X denotes one of the air pollutants including PM_10_, PM_2.5_, NO_2_, SO_2_, and O_3_-8 h.

We used the second levels of Air Quality Standard of China (GB3095-2012) as the references to estimate the influence of air pollution on varicella. There, the reference values for PM_10_, PM_2.5_, NO_2_, SO_2_, and O_3_-8 h were 70, 35, 40, 60, and 160 μg/m^3^, respectively^28^. Relative risks (RRs) were used to assess the lag-response relationships of single-day effects and cumulative effects per a 10-unit increase concentrations of the five pollutants. Besides, to explore the influence of extreme concentrations of air pollutants, we calculated the RRs and 95% confidence intervals (CIs) of varicella at the 2.5th and 97.5th percentiles of concentrations for different air pollutants relative to the class II levels of GB3095-2012 for separate and cumulative lag effect from 0 to 20 days.

We applied a series of sensitivity analyses to assess the robustness of the results: (1) fitting two or multi-pollutant models for all the air pollutants; (2) changing the df (6–8) per year for time. A two-sided *p* value ≤ 0.05 was regarded statistically significant. All analyses were performed based on R 3.6.3, and involved packages included “dlnm”, “mgcv”, and “splines”.

## Results

### Summaries of varicella cases, air pollutants, and meteorological factors

**Figure 1:**
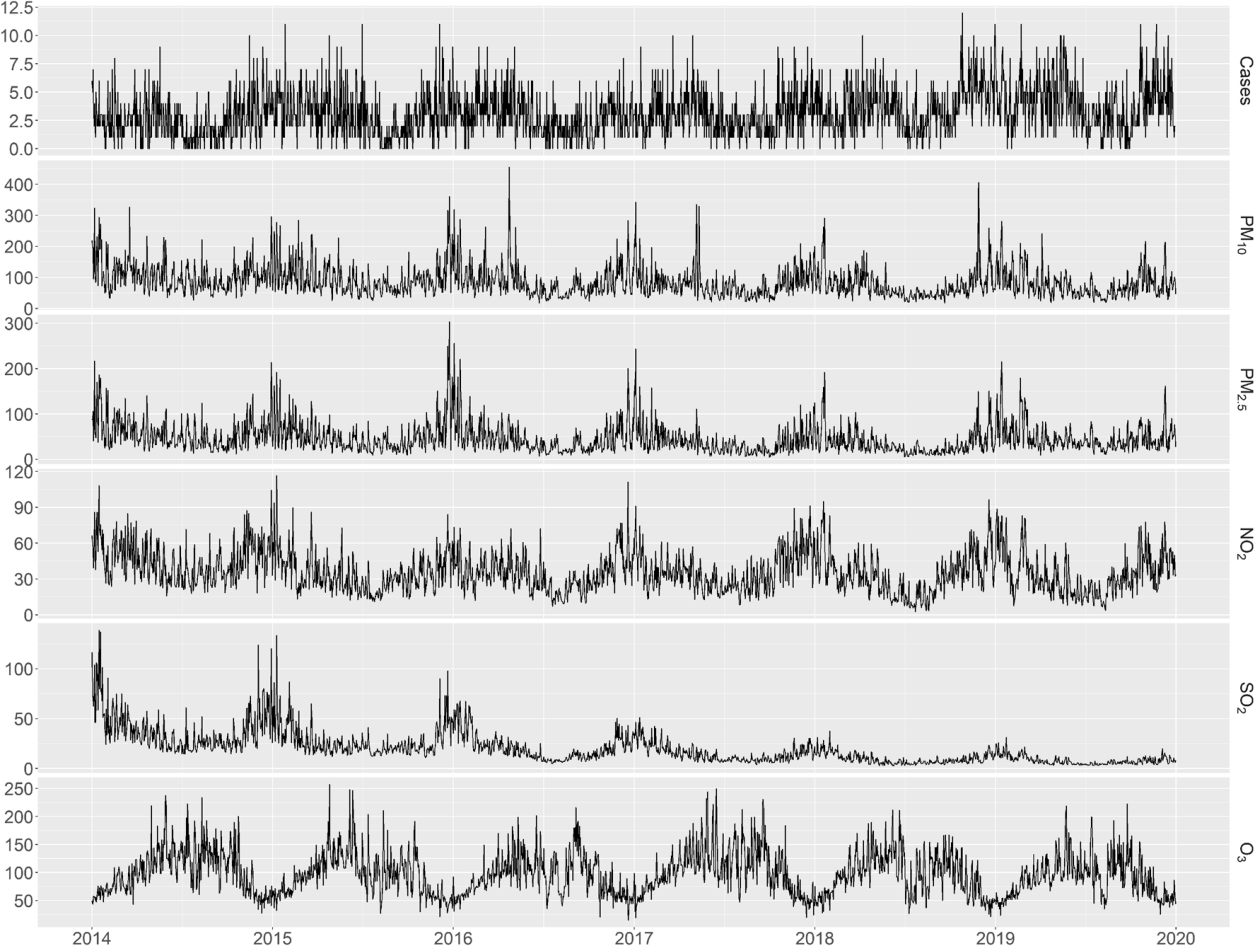
The temporal distributions of daily varicella cases and all air pollutants.

From 1 January 2014 to 31 December 2019, there were 2, 191 adult varicella cases reported in Qingdao. The female/male ratio was 0.919:1 (3331:3625). In Fig. 1, the season pattern of daily adult varicella cases took on an approximate double peak distribution in most years, which was similar to the season patterns found in Guangzhou^29^, Jinan^30^, Wuhan and Hongkong^31^. The peak of adult varicella cases occurred in November and December, and the smaller peak was found in April and May. The season patterns of air pollutants and meteorological factors were also shown in Fig. 1.

From 2014 to 2019, the daily mean content of PM_10_ was 86–68 μg/m^3^ (from 17.67 to 455.44 μg/m^3^), 45.7 μg/m^3^ for PM_2.5_ (4.38–304.11 μg/m^3^), 35.89 μg/m^3^ for NO_2_ (2.67–116.44 μg/m^3^), 19.74 μg/m^3^ for SO_2_ (2.56–138.78 μg/m^3^), and 97.57 μg/m^3^ for O_3_-8 h (14.88–257.22 μg/m^3^). The daily means of meteorological factors were 13.92 ℃ for temperature, 1.67 mm for cumulative precipitation, 1008.14 hPa for atmospheric pressure, 5.96 h for sunshine duration, 3.26 m/s for wind velocity, and 68.80% for relative humidity. More details were shown in Supplementary Table 1.

### Preliminary correlations

Table 1 indicated that daily contents of NO_2_, SO_2_, PM_10_, and PM_2.5_ were significantly related both to each other. O_3_ was significantly associated with NO_2_ and SO_2_. Besides, the five air pollutants were related to temperature, precipitation, relative humidity, and atmospheric pressure (*p* < 0.001). Furthermore, to control multicollinearity, we avoided including the pairs whose r ≥ 0.7 in our model simultaneously (temperature and atmospheric pressure: r = −0.84; PM_10_ and PM_2.5_: r = 0.90; PM_10_ and NO_2_: r = 0.71. All *p* < 0.001).

**Table 1:** Spearman’s correlation coefficient matrix between daily air pollutant concentrations and meteorological factors in Qingdao, 2014–2019.

	PM_10_	PM_2.5_	NO_2_	SO_2_	O_3_-8 h	Temperature	Precipitation	Atmospheric pressure	Sunshine duration	Wind velocity	Relative humidity
PM_10_	1										
PM_2.5_	0.90*	1									
NO_2_	0.71*	0.65*	1								
SO_2_	0.62*	0.56*	0.62*	1							
O_3_-8 h	0.01	−0.02	−0.20*	−0.14*	1	1					
Temperature	−0.35*	−0.38*	−0.46*	−0.43*	0.58*	1					
Precipitation	−0.33*	−0.25*	−0.25*	−0.23*	−0.10*	0.18*	1				
Atmospheric pressure	0.28*	0.26*	0.48*	0.37*	−0.54*	−0.84*	−0.28*	1			
Sunshine duration	0.10*	−0.02	0.04	0.07*	0.40*	0.17*	−0.46*	−0.08*	1		
Wind velocity	−0.02	−0.07*	−0.20*	−0.04	−0.14*	−0.25*	0.08*	0.08*	0.04	1	
Relative humidity	−0.31*	−0.12*	−0.48*	−0.32*	0.14*	0.41*	0.44*	−0.54*	−0.40*	−0.10*	1

*PM*_*10*_ particles with aerodynamic diameter less than 10 μm, *PM*_*2.5*_ particles with aerodynamic diameter less than 2.5 μm, *NO*_*2*_ nitrogen dioxide, *SO*_*2*_ sulfur dioxide, *O*_*3*_*-8 h* maximum 8-h moving average ozone concentration.

*p < 0.001.

### The associations between air pollutants and varicella

**Figure 2:**
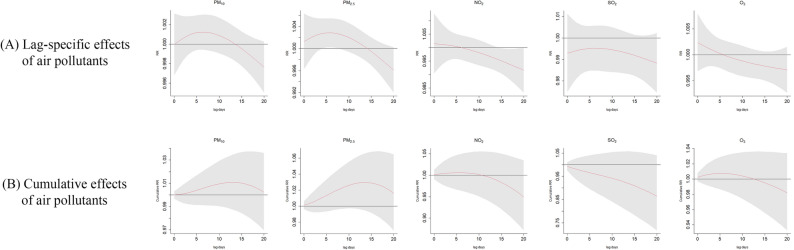
**(A)** Lag-specific RRs and **(B)** cumulative RRs in adult varicella cases per 10-unit increase in daily mean concentrations of air pollutants in the single-pollutant model; PM_10_, particles with aerodynamic diameter less than 10 μm; *PM*_*2.5*_ particles with aerodynamic diameter less than 2.5 μm, *NO*_*2*_ nitrogen dioxide, *SO*_*2*_ sulfur dioxide, *O*_*3*_ maximum 8-h moving average ozone concentration.

All the following results were shown in Fig. 2.

For a 10 μg/m^3^ increase of PM_10_, there were no significant associations in the lag-specific and cumulative effect curves. In lag-specific effect model, the RR was from 1.000 (95% CI: 0.997–1.003, lag day: 0) to the maximum value (RR: 1.001, 95% CI: 0.999–1.003, lag day: 6), and then it gone down to 0.998 (95% CI: 0.995–1.000, lag day: 20). In the cumulative effect curve, RR reached a maximum value with 1.011 (95% CI: 0.988–1.035, lag = 0–13 days).

Per increased 10 μg/m^3^ concentration of PM_2.5_ was significantly related to a higher risk of varicella from lag 4 day to lag 6 day (RR: 1.003, 95% CI: 1.000–1.005, lag day: 4; RR: 1.003, 95% CI: 1.000–1.005, lag day: 5; RR: 1.003, 95% CI: 1.000–1.005, lag day: 6). While in the cumulative effect curve, the association was non-significant. The RR rose to the highest value (RR: 1.030, 95% CI: 0.994–1.067, lag = 0–14 days).

Significant associations were also found in the relationship between the NO_2_ per 10 μg/m^3^ increase and the decreased risk of varicella from lag 15 day to lag 19 day (RR: 0.995, 95% CI: 0.990–0.999, lag day: 15; RR: 0.994, 95% CI: 0.989–0.999, lag day: 16; RR: 0.994, 95% CI: 0.988–0.999, lag day: 17; RR: 0.993, 95% CI: 0.987–0.999, lag day: 18; RR: 0.992, 95% CI: 0.985–0.999, lag day: 19). While in the cumulative effect curve, the association was non-significant. The RR first rose to the maximum value (RR: 1.006, 95% CI: 0.964–1.050, lag = 0–6 days), and then decreased gradually to 0.950 (95% CI: 0.871–1.035, lag = 0–20 days).

Per 10 μg/m^3^ increased concentration of SO_2_ was not significant related to varicella among adults not only in lag-specific effect curve but also in the cumulative effect model. At lag 6 day, in lag-specific effect model, the RR reached the peak value (RR: 0.955, 95% CI: 0.985–1.006). And in cumulative effect curve, RR gradually decreased to 0.864 (95% CI: 0.717–1.040, lag = 0–20 days).

For per 10 μg/m^3^ increase of O_3_ concentration, there were no significant associations between O_3_ and varicella incidence both in lag-specific effect model and in cumulative effect curve. In cumulative effect curve, RR gradually decreased from 1.002 (95% CI: 0.997–1.008; lag day: 0) to 0.997 (95% CI: 0.993–1.002; lag day: 20). In cumulative effect curve, RR increased to the peak value (RR: 1.008; 95% CI: 0.987–1.029; lag day: 5), and then decreased.

### The effects of extreme concentrations of air pollutants on varicella

**Figure 3:**
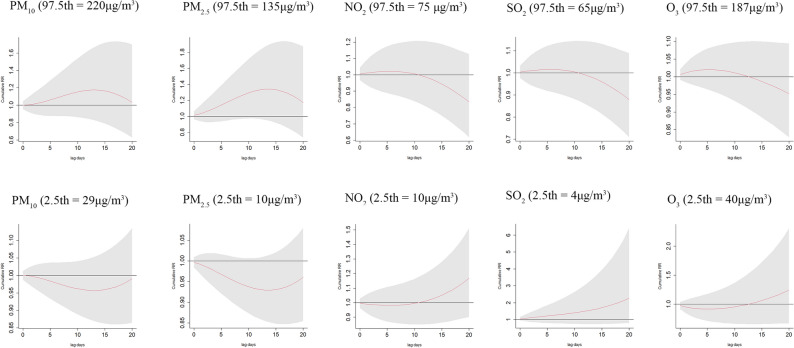
The cumulative effects of extreme PM_10_, PM_2.5_, NO_2_, SO_2_, O_3_ on the incidence of varicella.

The effects of extreme contents of air pollutants on varicella at each single lag days were shown in Table 2. When compared with 35 μg/m^3^, the 97.5th percentile of PM_2.5_ concentration was significantly related to the increased risk of varicella on lag 6 day and lag 7 day (the RRs were both 1.026, 95% CI: 1.000–1.053). For NO_2_, compared with 40 μg/m^3^, the significant associations were found between both at 97.5th and 2.5th percentiles of concentration and varicella on lag 15 day to lag 20 day. The more details were shown in Table 2. However, there were no significant associations found in the cumulative effects of extreme PM_10_, PM_2.5_, NO_2_, SO_2_, O_3_ on varicella (Fig. 3).

**Table 2:** The distributed lag effects of extreme PM_10_, PM_2.5_, NO_2_, SO_2_, O_3_ concentrations on varicella at various lag days.

Lag	PM_10_		PM_2.5_		NO_2_		SO_2_		O_3_	
	97.5th (220 μg/m^3^)	2.5th (29 μg/m^3^)	97.5th (135 μg/m^3^)	2.5th (10 μg/m^3^)	97.5th (75 μg/m^3^)	2.5^th^ (10 μg/m^3^)	97.5th (65 μg/m^3^)	2.5th (4 μg/m^3^)	97.5th (187 μg/m^3^)	2.5th (40 μg/m^3^)
1	0.998 (0.953, 1.045)	1.001 (0.988, 1.013)	1.011 (0.962, 1.062)	0.997 (0.985, 1.010)	1.000 (0.963, 1.039)	1.000 (0.968, 1.033)	0.996 (0.987, 1.005)	1.042 (0.942, 1.152)	1.006 (0.992, 1.021)	0.972 (0.910, 1.038)
2	1.002 (0.964, 1.043)	0.999 (0.989, 1.010)	1.015 (0.974, 1.058)	0.996 (0.986, 1.007)	1.001 (0.970, 1.032)	1.000 (0.973, 1.026)	0.997 (0.989, 1.004)	1.039 (0.953, 1.132)	1.005 (0.993, 1.017)	0.978 (0.926, 1.032)
3	1.007 (0.973, 1.041)	0.998 (0.989, 1.007)	1.020 (0.985, 1.056)	0.995 (0.987, 1.004)	1.001 (0.976, 1.026)	0.999 (0.978, 1.021)	0.997 (0.990, 1.003)	1.036 (0.962, 1.115)	1.004 (0.994, 1.014)	0.983 (0.940, 1.028)
4	1.010 (0.981, 1.041)	0.997 (0.989, 1.005)	1.023 (0.992, 1.054)	0.994 (0.987, 1.002)	1.000(0.980, 1.021)	1.000 (0.982, 1.017)	0.997 (0.991, 1.003)	1.033 (0.968, 1.103)	1.003 (0.994, 1.011)	0.988 (0.951, 1.026)
5	1.013 (0.985, 1.041)	0.997 (0.989, 1.004)	1.025 (0.997, 1.053)	0.994 (0.987, 1.001)	1.000 (0.983, 1.018)	1.000 (0.985, 1.015)	0.997 (0.992, 1.003)	1.031 (0.970, 1.096)	1.002 (0.994, 1.009)	0.993 (0.959, 1.028)
6	1.015 (0.987, 1.042)	0.996 (0.989, 1.003)	1.026 (1.000, 1.053)	0.994 (0.987, 1.000)	0.999 (0.983, 1.016)	1.001 (0.987, 1.015)	0.997 (0.992, 1.003)	1.030 (0.971, 1.093)	1.001 (0.993, 1.008)	0.997 (0.964, 1.031)
7	1.015 (0.988, 1.043)	0.996 (0.988, 1.003)	1.026 (1.000, 1.053)	0.994 (0.987, 1.000)	0.998 (0.983, 1.014)	1.001 (0.988, 1.015)	0.997 (0.992, 1.003)	1.030 (0.970, 1.093)	1.000 (0.992, 1.007)	1.001 (0.968, 1.036)
8	1.015 (0.988, 1.044)	0.996 (0.988, 1.003)	1.026 (0.999, 1.053)	0.994 (0.987, 1.000)	0.997 (0.981, 1.013)	1.003 (0.989, 1.016)	0.997 (0.992, 1.003)	1.030 (0.970, 1.093)	0.999 (0.991, 1.007)	1.005 (0.971, 1.04)
9	1.014 (0.987, 1.043)	0.996 (0.989, 1.004)	1.024 (0.997, 1.051)	0.994 (0.988, 1.001)	0.996 (0.980, 1.012)	1.004 (0.990, 1.018)	0.997 (0.992, 1.003)	1.030 (0.970, 1.094)	0.998 (0.990, 1.006)	1.008 (0.974, 1.045)
10	1.013 (0.985, 1.042)	0.996 (0.989, 1.004)	1.022 (0.995, 1.049)	0.995 (0.988, 1.001)	0.994 (0.978, 1.010)	1.005 (0.991, 1.019)	0.997 (0.992, 1.003)	1.031 (0.971, 1.095)	0.997 (0.989, 1.005)	1.012 (0.976, 1.048)
11	1.011 (0.983, 1.039)	0.997 (0.990, 1.005)	1.019 (0.992, 1.046)	0.995 (0.989, 1.002)	0.992 (0.976, 1.009)	1.007 (0.993, 1.021)	0.997 (0.992, 1.003)	1.033 (0.972, 1.096)	0.997 (0.989, 1.005)	1.015 (0.980, 1.051)
12	1.008 (0.981, 1.036)	0.998 (0.990, 1.005)	1.015 (0.989, 1.042)	0.996 (0.990, 1.003)	0.990 (0.974, 1.006)	1.009 (0.995, 1.023)	0.997 (0.992, 1.002)	1.034 (0.975, 1.097)	0.996 (0.988, 1.004)	1.017 (0.983, 1.053)
13	1.005 (0.978, 1.032)	0.999 (0.991, 1.006)	1.011 (0.985, 1.037)	0.997 (0.991, 1.004)	0.988 (0.972, 1.004)	1.010 (0.997, 1.024)	0.997 (0.992, 1.002)	1.037 (0.978, 1.098)	0.996 (0.988, 1.003)	1.020 (0.986, 1.055)
14	1.001 (0.974, 1.028)	1.000 (0.993, 1.007)	1.006 (0.981, 1.031)	0.999 (0.992, 1.005)	0.986 (0.970, 1.001)	1.012 (0.999, 1.026)	0.997 (0.992, 1.002)	1.039 (0.982, 1.099)	0.995 (0.988, 1.002)	1.022 (0.989, 1.057)
15	0.997 (0.971, 1.023)	1.001 (0.994, 1.008)	1.001 (0.976, 1.026)	1.000 (0.994, 1.006)	0.983 (0.968,0.999)	1.015 (1.001, 1.028)	0.996 (0.991, 1.001)	1.042 (0.986, 1.101)	0.995 (0.987, 1.002)	1.024 (0.991, 1.059)
16	0.992 (0.966, 1.018)	1.002 (0.995, 1.009)	0.995 (0.970, 1.020)	1.001 (0.995, 1.008)	0.981 (0.965,0.997)	1.017 (1.003, 1.031)	0.996 (0.991, 1.001)	1.045(0.990, 1.103)	0.994 (0.987, 1.002)	1.026 (0.993, 1.061)
17	0.987 (0.961, 1.014)	1.004 (0.996, 1.011)	0.989 (0.964, 1.015)	1.003 (0.996, 1.009)	0.978 (0.962,0.995)	1.019 (1.004, 1.034)	0.996 (0.991, 1.001)	1.048(0.992, 1.108)	0.994 (0.986, 1.002)	1.028 (0.993, 1.065)
18	0.982 (0.955, 1.010)	1.005 (0.997, 1.013)	0.983 (0.956, 1.010)	1.004 (0.998, 1.011)	0.975 (0.957,0.994)	1.022 (1.005, 1.038)	0.995 (0.990, 1.001)	1.052 (0.994, 1.113)	0.993 (0.985, 1.002)	1.030 (0.992, 1.071)
19	0.976 (0.948, 1.006)	1.007 (0.998, 1.015)	0.976 (0.947, 1.006)	1.006 (0.999, 1.014)	0.973 (0.953,0.993)	1.024 (1.006, 1.042)	0.995 (0.990, 1.001)	1.056 (0.994, 1.121)	0.993 (0.983, 1.002)	1.032 (0.989, 1.077)
20	0.971 (0.940, 1.003)	1.008 (0.999, 1.017)	0.970 (0.938, 1.002)	1.008 (0.999, 1.016)	0.970 (0.948,0.993)	1.026 (1.006, 1.047)	0.995 (0.989, 1.001)	1.060 (0.993, 1.131)	0.993 (0.982, 1.003)	1.034 (0.986, 1.084)

PM_10_, particles with aerodynamic diameter less than 10 μm; PM_2.5_, particles with aerodynamic diameter less than 2.5 μm; NO_2_, nitrogen dioxide; SO_2_, sulfur dioxide; O_3_, maximum 8-h moving average ozone concentration.

### Robustness of DLNM

The results were robust after applying multi-pollutant models. (Supplementary Fig. 2) When changing the df of seasonality and long-time trend, the results remained broadly robust (Supplementary Fig. 3).

## Discussion

The results from DLNM showed that exposure to PM_2.5_ and NO_2_ were significantly associated with the risk of varicella among adults. To the best of our knowledge, this is the first study analyzing the association between PM_10_, PM_2.5_, NO_2_, SO_2_, O_3_ and varicella. Besides, our study focused on adults.

Our results implied that PM_2.5_ may result in a higher risk of varicella. Firstly, mucus on the epithelial surface of nasal cavity and respiratory tract can prevent the invasion of the virus. Exposure to air pollution may interfere with the secretion of the normal clearance of the virus, which could help the virus escape the first level of defense^32^. Secondly, particulate matter have the ability to adhere to virus, making them good carriers of the virus^33,34^. Aerosols with aerodynamic diameter ≤ 2.5 μm can be transmitted over a long distance by air flow, therefore the virus could also spread a long distance^35^. Thirdly, PM_2.5_ can absorb impurities on its surface, such as volatile organic compounds and heavy metals^36,37^, thus lead to oxidative stress. For one thing, oxidative stress could mediate inflammation^38,39^, thus damaging the immune functions of the cells^40,41^. For another, oxidative stress may cause mutation of gene expression, further resulting in sensitivity to respiratory infection of the body^20^.

Exposure to high concentration of NO_2_ (at 97.5th percentile) showed a protective effect on the incidence of varicella. As we all know, NO_2_ was widely used as disinfectant in treatment of industrial water. This reminds us that NO_2_ may inhibit the spread of the virus attribute to its oxidability.

In our study, no significant association was found between PM_10_ and varicella. However, there was only one research assessing the relationship between coarse particulate matter and varicella conducted by Yu et al., which found that high PM_10_ concentration (> 300 μg/m^3^) could result in a higher risk of varicella^19^. The possible causes for this heterogeneity were as follows. Firstly, Yu’s study was conducted in Jiading District, Shanghai but our research was in Qingdao, Shandong province. The difference of geographical areas results in the divergence of meteorological variables, such as temperature, humidity, wind speed and etc. Secondly, the levels of PM_10_ concentrations were different in these two cities. The maximum concentration of PM_10_ in Shanghai was higher than that in Qingdao (Shanghai: 600.00 μg/m^3^; Qingdao: 455.44 μg/m^3^). And in Qingdao, there was only 12 days during 2014–2019 when the concentration of PM_10_ exceeded 300 μg/m^3^. Finally, Yu’s research carried out in the whole population, while we only focused on the effects among adults.

Some strengths were in our study. Firstly, this is the first study assessing the associations between several air pollutants (PM_10_, PM_2.5_, NO_2_, SO_2_, and O_3_) and the incidence of varicella. Besides, given severe complications and even death can occur among adults, our research focused on adults was necessary and important. Secondly, we explored the influence of exposure to extremely high and low concentrations of air pollutants. Thirdly, our study provided evidence for public health officials to establish a mature environmental monitoring system to predict epidemics of varicella among adults.

However, some limitations were also found in our study. Firstly, our findings were subject to ecological fallacies. In ecological study, all individuals were supposed to be exposed to the same contents of air pollutants. And causal relationship is difficult to determine, there is often an accompanying relationship between the two variables. Secondly, this study was limited to Qingdao, which may affect the extrapolation of the results. More studies especially multi-city studies are needed to confirm the results. Thirdly, the effect of air pollution is a total effects of various air pollutants. However, we only analyzed PM_10_, PM_2.5_, NO_2_, and SO_2_, not including other air pollutants. Besides, there is no good way to explore the mixtures effects of air pollutants. Finally, other host and environmental factors which have the significant impacts on the epidemic of varicella were not included in our study, such as population density, host susceptibility, and human serum antibody data.

## Conclusions

Exposure to PM_2.5_ was associated with the increased risk of varicella. And the significant relationship was found between exposure to NO_2_ and the incidence of varicella. This might provide evidence for health authorities to establish a mature environmental monitoring system for varicella among adults.

## Supplementary Information

Supplementary Information.

## Data Availability

The data used and/or analyzed in the current study are not publicly available because restrictions apply to the availability of these data. Government departments allow researchers to use the data for scientific research, but do not allow anyone to share original data publicly. If other researchers need the data used in this study, please apply to Qingdao Municipal Center for Disease Control and Prevention. Data are available from the corresponding author on reasonable requests and with permission of Qingdao Municipal Center for Disease Control and Prevention.
