# Supplementary material for: Influence of air pollutants on varicella among adults
Source: Sci Rep. 2021 Oct 25;11:21020. doi: 10.1038/s41598-021-00507-z (PMC8546085; doi:10.1038/s41598-021-00507-z)
Supplement: Supplementary file 1 — Supplementary Information. [file 41598_2021_507_MOESM1_ESM.pdf]

Influence of air pollutants on varicella among adults

Zixuan Wang [a,1], Xiaofan Li [b,1], Ping Hu [b], Shanpeng Li [b], Jing Guan [b], Bingling Wang [b], Feng Yang [b,*] ,Dongfeng Zhang [a,**]

[a] Department of Epidemiology and Health Statistics, The School of Public Health of Qingdao University, Qingdao, 266021, Shandong Province, China.

[b] Qingdao Municipal Center for Disease Control and Prevention of Qingdao, Qingdao Institute of Preventive Medicine, Qingdao, Shandong, 266034, China.

[1] The authors contribute equally.

[*] Corresponding author.

[**] Corresponding author at: Department of Epidemiology and Health Statistics, The School of Public Health of Qingdao University, No. 308 Ningxia Road, Qingdao 266021, China.

E-mail addresses: xiaoxianer6@163.com(F.Yang) ;

zhangdf1961@126.com (D.Zhang).

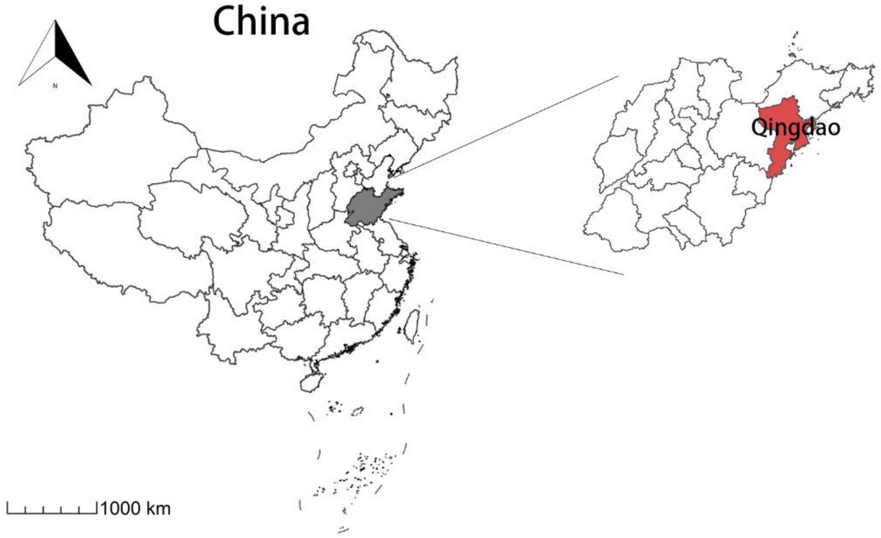

Supplementary Figure 1 The geographical location of Qingdao, China. (created by R 3.6.3,

URL: https://mirrors.sjtug.sjtu.edu.cn/cran/)

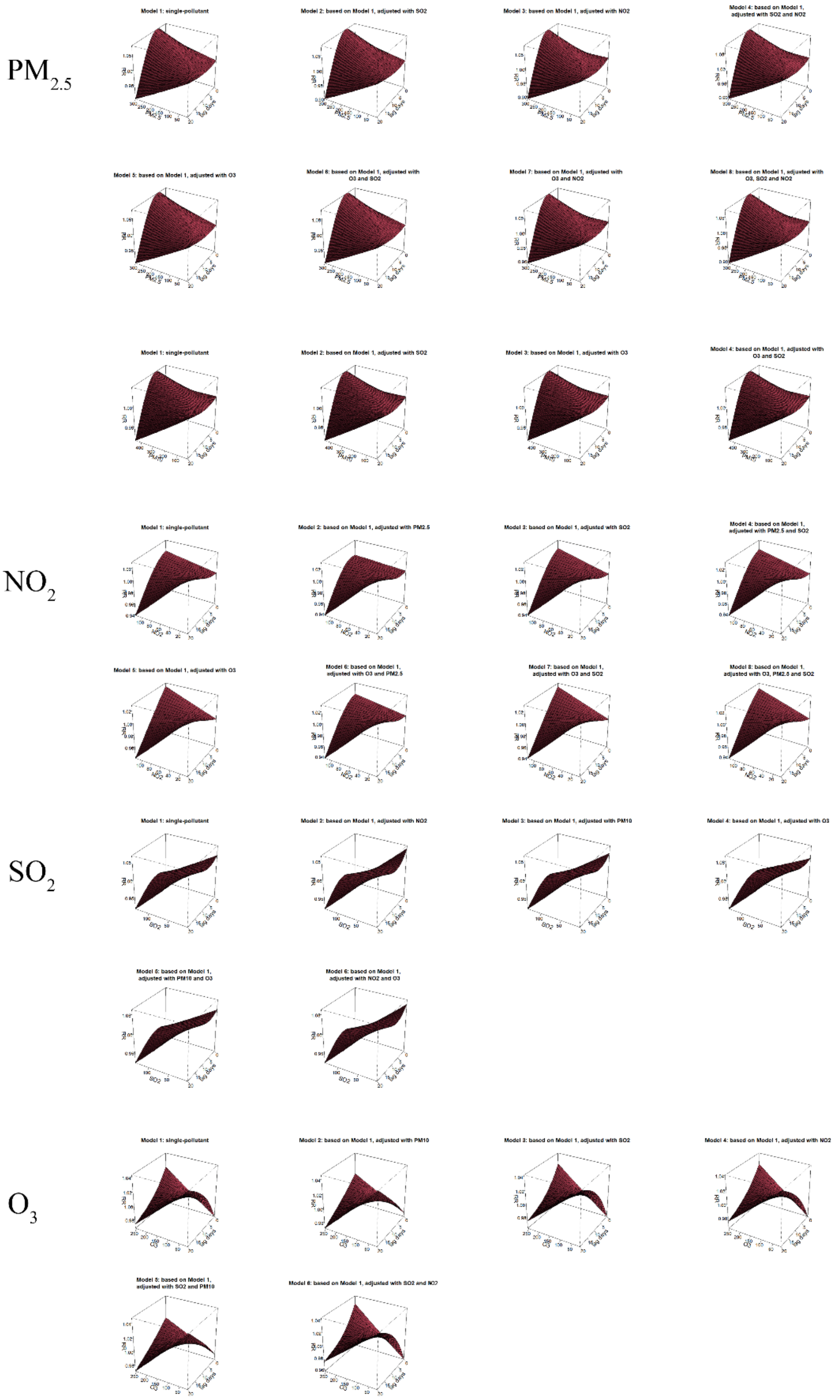

Supplementary Figure 2 Results of sensitive analysis by conducting multi-pollutant models.

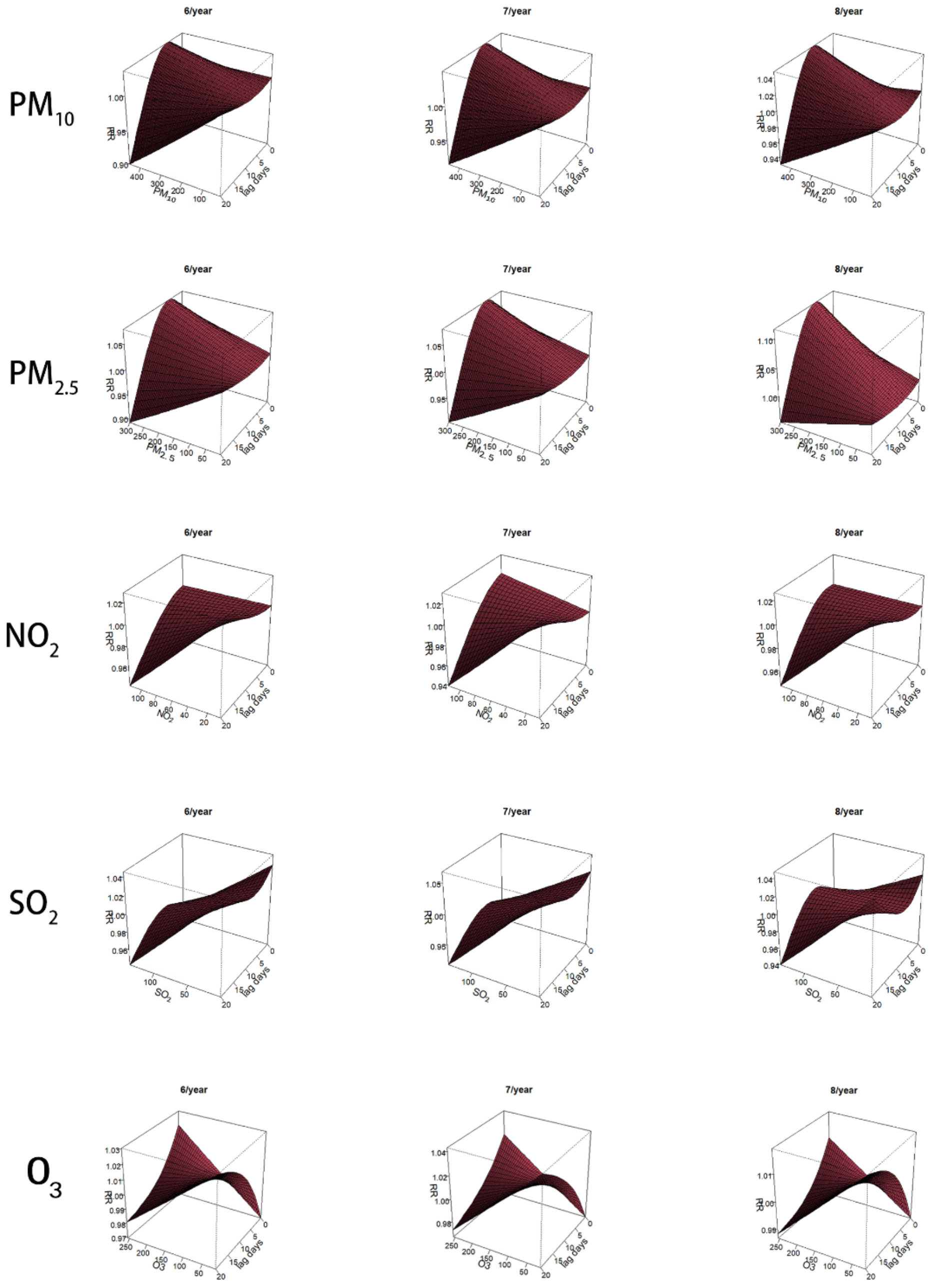

Supplementary Figure 3 Results of sensitive analysis by changing the degree of freedom of long-term trend and seasonality in single-pollutant models.

Supplementary Table 1. Summary of daily air pollutant concentrations, meteorological factors and varicella cases in Qingdao, 2014-2019.

Variables	Mean	Std.deviation	Percentile			Min	Max
			25	50	75		
Cases	3.17	2.15	2.00	3.00	4.00	0.00	12.00
Pollutant concentration							
PM_{10} (μg/m^3)	86.68	50.31	52.17	73.62	107.53	17.67	455.44
$PM_{2.5}$ (μg/m^3)	45.70	33.21	23.44	36.25	57.78	4.38	304.11
NO_2 (μg/m^3)	35.89	17.14	23.33	33.00	46.00	2.67	116.44
SO_2 (μg/m^3)	19.74	16.55	8.33	15.43	25.00	2.56	138.78
O_3-8h (μg/m^3)	97.57	38.90	66.94	94.33	130.94	14.88	257.22
Meteorological factors							
Temperature (℃)	13.92	9.21	5.70	14.70	22.20	-11.50	30.60
Precipitation (mm)	1.67	7.39	0	0	0	0	129.90
Atmospheric pressure (hPa)	1008.14	9.07	1000.40	1008.20	1015.40	979.30	1032.30
Sunshine duration (h)	5.96	3.80	2.60	6.90	9.00	0	13.00
Wind velocity (m/s)	3.26	1.34	2.30	3.00	3.90	0.90	10.00
Relative humidity (%)	68.80	16.41	56.00	70.00	83.00	16.00	100.00
